# An Exploratory Investigation Into Women's Experience of Sexual Harassment in the Workplace

**DOI:** 10.1177/10778012221114921

**Published:** 2022-08-15

**Authors:** Ashleigh Spiliopoulou, Gemma L. Witcomb

**Affiliations:** 1 School of Sport, Exercise and Health Sciences, 5156 Loughborough University, Loughborough, UK

**Keywords:** harassment, gender, thematic analysis, online

## Abstract

The recent surge in online movements challenging the culture of silence surrounding sexual harassment has created new spaces for women to share their stories. This research employed a qualitative, exploratory design to study 199 comments on a public online community forum: “What's The Wildest Thing That Happened To You As A Working Woman?”. Inductive thematic analysis was performed on the data which resulted in three overarching themes: *“a harassment endemic,” “the (im)balance of power,”* and *“it's in the culture”.* Sexual harassment was centered as a normal part of women's workplace experience, as was lack of affirmative action from employers which increased the severity of experiences. Organizations must commit to challenging the structures and individuals that perpetuate unsafe working conditions for women.

## Introduction

The prevalence of sexual harassment experienced by women has gained increasing attention in the media over recent years. While the circumstances under which sexual harassment is experienced are widespread and varied, the gender of the victims is not.

Workplace sexual harassment is sadly common and gendered. For example, Fitzgerald and Cortina (2018) estimated that half of all working women will experience sexual harassment during their working life and polling by the Trades Union Congress (2016) reported that women are 3 times more likely than men to be a victim. However, a 2021 UN survey found that 80% of sexual harassment victims in the UK never report the incident (UN Women UK, 2021) and such a significant number of unreported cases makes it difficult to obtain precise prevalence figures. Indeed, a culture of non-reporting/non-reacting underlies the narrative that workplace sexual harassment is uncommon.

There are competing legal and sociopsychological definitions of sexual harassment. The legal profession requires objectivity whereas the sociopsychological standpoint is based on the victim's interpretation of their experience. From the sociopsychological perspective, sexual harassment refers to “unwanted conduct of a sexual nature which has the purpose or effect of violating someone's dignity or creating an intimidating, hostile, degrading, humiliating or offensive environment for them” (Trades Union Congress, 2016). In order to understand what this definition looks like in terms of behavior, Fitzgerald and Cortina (2018) created three categories of sexual harassment based on a review of the previous literature. *Gender harassment* was the most common experience and refers to hostile or degrading attitudes about women. *Unwanted sexual attention* encompassed any unwelcome or uninvited sexual advances. Finally, *sexual coercion* referred to sexual advances made specifically in exchange for some benefit or threat of negative consequence. Fundamentally, however, all three types of sexual harassment have significant negative consequences for women; for their mental health, physical health, and opportunities within society. For example, the psychological stress of coping increases the likelihood of suffering from anxiety, depression, or insomnia (e.g., Houle et al., 2011; Walker, 2018), with the risk of developing post-traumatic stress symptoms exacerbated by remaining silent about one's experience (Baum, 2019). Physically, research has shown that women who experience harassment are at greater risk of adverse physiological responses, such as raised blood pressure, headaches, and gastrointestinal distress (Baum, 2019). Finally, experiences of harassment can directly impact career progression and job exit (McLaughlin et al., 2017) with women who report harassment being less likely to be recommended for promotion (Hart, 2019). Thus, women are subject to inequity of opportunity due to misogynistic working environments. Aside from the moral and legal obligations of organizations to address harassment in the workplace, these outcomes should also be of critical concern for them since poor job performance and premature resignation impacts the productivity of a company as a whole (Walker, 2018). However, due to the vast underreporting many companies may be unaware of the problem or—potentially more probable—uncomfortable in drawing attention to it (Hershcovis et al., 2021).

Organizational culture is largely responsible for the silence surrounding workplace sexual harassment. Though it would seem to be in an organization's interest to challenge sexual harassment, many women believe that their employer would not support them if they were to report an incident. Worryingly, this perception is justified. Walker (2018) found that 72% of reported sexual harassment cases ended in the employer condoning, denying, or rationalizing the incident. Even more concerning, many women were deterred from reporting sexual harassment because they feared retaliation from their employer, having seen others face adverse employment and social consequences as a result of reporting (Walker, 2018). Often retaliation consisted of tangible employment consequences such as termination or the denial of promotional opportunities. However, it could also take the form of social retaliation. Brown and Battle (2019) found that women who reported sexual harassment were ostracized within the workplace, which was highly damaging to their self-esteem and sense of belonging at work. The researchers concluded that social retaliation was both a consequence of, and a significant barrier to, reporting sexual harassment.

Other researchers have sought to understand the motivation behind sexual harassment. A number of theories have been suggested to try to explain workplace sexual harassment (Kapila, 2017). Socio-cultural theories view harassment as a product of the wider gender inequalities that exist in society. For example, sexual harassment in the military is common and women report being regarded by males as disrupting male cohesion in a culture that is organized around male bonding and male superiority and power (Pershing, 2003). Organizational theory relates harassment to power dynamics stemming from hierarchical structures. Indeed, there are high incidences of sexual harassment within industries that have a significant gender disparity in the balance of power within their hierarchies, but also in the service sector, where there is a power disparity between the employee and the customer. Klein et al. (2020) have suggested that many service sector organizations prioritize customer satisfaction over staff well-being due to the essential role customers play in the survival of the industry. Consequently, the researchers concluded that tolerating sexual harassment has become a condition of employment for many service sector staff and is often regarded as necessary to earn tips, rendering them powerless to report it if they want to continue working in the industry.

Despite the progress that has been made in understanding workplace sexual harassment, the culture of silence surrounding it has adversely affected research development. It has been difficult to gain a deep insight into the victim's perspective on sexual harassment because many women are afraid to disclose their stories due to a fear of retaliation or because they are uncertain as to whether their experience can be considered sexual harassment (Freedman-Weiss et al., 2020). However, in recent years, an online community has emerged to oppose the isolation victims of workplace harassment were experiencing at work. In 2017, Alyssa Milano tweeted “if you’ve been sexually harassed or assaulted write ‘me too’ as a reply to this tweet.” The #MeToo movement demonstrated the magnitude of a problem that had been masked by mass underreporting, with Facebook recording 4.7 million users of the hashtag within 24 h of Milano's tweet (Baum, 2019). #MeToo created a space for female victims to share their experiences without fear of retaliation, disbelief, or judgement. Not only has this demonstrated the vast range of experiences which constitute sexual harassment but it has also begun to dismantle the normalization of these abusive behaviors. More recently, Soma Sara launched the Everyone's Invited movement after her Instagram post in which she had shared her personal experience of rape received hundreds of responses from women who felt her experience resonated with their own. Online movements such as #MeToo and Everyone's Invited demonstrate that online platforms can be used to unite, support, and educate communities about sexual harassment.

Furthermore, these movements have created a rich and extensive online dataset of female testimonies which remain largely untapped by previous researchers. The present study aims to utilize this valuable data by studying women's experiences of workplace sexual harassment using data from an online community forum. Online data of this kind is valuable because it was not created for research purposes and as such it is exempt from the limitations pertaining to potential psychological harm, researcher bias, and social desirability effects that are involved in traditional qualitative interview studies. As a result it has the potential to offer a deeper, more candid insight into women's experiences of workplace sexual harassment. It was important that the data should be allowed to drive this study which is why an inductive approach was taken and no specific hypotheses were made. The aim of the study is to expand upon researchers’ understanding of any aspect of women's experience of workplace sexual harassment, which could include information on the victims, perpetrators, organizations, and circumstances involved.

## Methods

This was a qualitative study of reader comments made on an online community thread about women's experiences in the workplace. All of the data used in this study is publicly available on the Buzzfeed website. Previous research using reader comments have produced findings that reflect those found in other forms of qualitative data suggesting that reader comments are “transferable” (Thomas-Meyer et al., 2017).

### Ethics and Consent

This study was granted ethical approval from Loughborough University and adheres to the British Psychological Society guidelines. Data consisted of comments which were freely posted on a publicly available, open-access website. Consequently, the data collection process is akin to behavioral observation in a public space where individuals could reasonably expect to be observed. Care was taken to anonymize commenters by omitting usernames from any quotations used within the research.

### Data Collection

The data was collected from a single thread on the Buzzfeed website: “What's The Wildest Thing That Happened To You As A Working Woman?” (Gant, 2017) from November 2017 to March 2020. Buzzfeed users had to create a Buzzfeed account and a username before they could comment. Users could like and reply to previous comments which allowed conversations to develop between users. The original thread contained a total of 230 comments ranging in length from one word to several hundred. The majority of users posted once although some users posted multiple comments. This study was specifically interested in comments about sexual harassment rather than other workplace experiences. As a result, all of the comments were read and any comments unrelated to sexual harassment were removed. Only 30 comments were removed for this reason. In addition, one comment was removed because its content had been deleted from the original thread. This left 199 comments for analysis.

### Analyses

The comments were collected on November 29, 2020. The data was analyzed using thematic analysis in line with Braun and Clarke’s (2006) six-step methodology. In order to allow the data to drive the analysis, an inductive approach was taken. The first stage of the analytic process involved an in-depth reading of the data by the first researcher. After the data had been read and re-read to ensure full comprehension, it was coded by hand on a line-by-line basis. The codes were then grouped into 40 preliminary descriptive themes. These themes were reviewed and modified by re-reading the data before being grouped into eight broader themes. Continuously returning to the data throughout the analytic process ensured that the themes remained data driven and that the extracts were illustrative of the corresponding theme. The aim was to ensure that the themes were distinctive from each other and that each of them had explanatory value. After reviewing the themes in comparison with the whole dataset, some themes were combined and others removed because they did not adequately capture the extracts within them. Coding was completed when the researcher was satisfied that the themes accurately represented the data. In the end, three overarching themes were identified, named, and defined. Subthemes were used within each theme to help structure the meaning of the data. Each data extract was labeled with a numerical pseudonym such as U1 which denoted which user the extract came from. This enabled the researcher to see when multiple extracts came from the same user.

## Results

Some women wrote at length about their experience of workplace sexual harassment while other comments were more succinct. An average comment was around 200 words. The data confirmed the findings of previous research in terms of the type of sexual harassment women experience at work (Fitzgerald & Cortina, 2018). Gender harassment, unwanted sexual attention, and sexual coercion were all experienced by women who commented on the thread, with some women experiencing multiple kinds of sexual harassment. Rather than reiterating these findings, this study concentrates on women's experience of sexual harassment as systemic within the workforce, driven by a framework of power, and upheld by organizational cultures which ignore, tolerate, or accept harassment.

The first theme, *a harassment endemic,* includes stories from women who experienced sexual harassment as a normal part of working life. The second theme, *the (im)balance of power,* refers to the narrative of dominance thar surrounded women's experiences of sexual harassment. The final theme, *it's in the culture,* explores how ineffective organizational responses impacted women's experience of sexual harassment. Within each theme there were a number of subthemes, amounting to 10 subthemes in total. Grammar and spelling errors have been maintained in order to preserve the integrity of the experiences.

### A Harassment Endemic

Within this theme, women described how sexual harassment was part of being a working woman. The sentiment among many of the women was that sexual harassment had become normal within the workplace because it happened so regularly and because men condoned sexually harassing behavior. The different subthemes explore the factors which contributed to the normalization of sexual harassment as part of the female working experience.

## An Everyday Occurrence

Some women gave the impression that they had become numb to sexual harassment because of how regularly they experienced it. Rather than expressing shock or horror, women often used phrases such as *“I can’t even tell you the number of times” (U1)* and *“I was harassed all the time” (U2)* which suggests they had become resigned to the fact that sexual harassment was a constant part of their working experience. This point was emphasized by women who described sexual harassment as an unrelenting experience through the use of specific temporal markers, such as *“daily” (U3)* and *“this went on for six months” (U4)*. One woman provided further explanation of the way that sexual harassment had become usual, typical, and expected:

These experiences, including unwanted sexual touching, him removing his clothing in front of me, crude comments or inappropriate requests or propositions, have happened multiple times to me, by multiple employers. I know I’m not even close to the only women whose experienced these things. These experiences or similar ones are a chapter, that I have simply titled, “A Working Woman” that exists in the stories of many women's lives, unfortunately. (U5)

The metaphor of her life as a book, of which sexual harassment is one chapter, helps this woman to convey that she has internalized sexual harassment as part of her identity as a working woman. She is resigned to sexual harassment being “part of the job” for women who enter the workforce. The fact that sexual harassment has become entwined within her life story demonstrates that she has taken on the burden of it, despite being the victim. The suggestion is that she experiences sexual harassment as her issue, which is further emphasized by the fact that the perpetrator remains anonymous and thus detached from it. She goes on to generalize her experience to other women by anonymizing the chapter *“A Working Woman.”* The implication is that rather than being an issue of personal lust or attraction, one's status as a working woman is criteria enough to expect to experience sexual harassment at work.

## Does No Mean No?

This subtheme refers to comments from women who were made to feel that it was unacceptable to reject male sexual advances at work. These women were threatened or ridiculed by men when they challenged sexually harassing behavior, which contributed to the normalization of workplace sexual harassment.

I repeatedly asked my former boss to stop putting his hands around my waist and on the small of my back and he not only refused, but then complained about my request for bodily autonomy to other male employees, who sided with him and harassed me about it. (U6)

Her objection to unconsented touching is deemed by the male employees to be more offensive than the act of touching a woman without consent. By siding with the perpetrator, the men imply that they feel it is acceptable for him to have touched a female colleague without asking her. In contrast, their further harassment of her suggests that they consider her request for bodily autonomy to be abnormal and intolerable. By ostracizing and targeting a woman who stands up to sexual harassment, her behavior is problematized while the perpetrator's harassing behavior is normalized.

In other cases, women were verbally abused and threatened for resisting sexual harassment. In one case “a male co-worker followed me into an elevator and tried to make out with me. When I pushed him away he called me a Bitch” (U7) while another woman was “threatened with stalking for not giving my number to a patron” (U8). By challenging harassment, these women were subjected to further abuse. The fact that their resistance did not deter men from harassing them demonstrates that men do not perceive sexual harassment to be problematic behavior. The men's reaction implies that they consider it to be their right to proposition women at work and that it is unreasonable for their advances to be rejected. By verbally harassing women who challenge sexual harassment, men are prioritizing their ego over female bodily autonomy and promoting the view that sexual harassment is a normal and acceptable workplace practice. Women who object to it are considered abnormal and exposed to further abuse.

### The (Im)Balance of Power

In this theme, women discussed how workplace power dynamics affected their experience of sexual harassment. In many comments, the perpetrator held a position of dominance over the victim which made it difficult for the victim to challenge them. The different subthemes represent the different forms of dominance that men utilized to sexually harass women in this sample.

## The Abuse of Authority

The first subtheme, *the abuse of authority*, refers to comments where women were harassed by men in senior, managerial positions. One woman described how:

the other admin at the office told me he invited her to go with him the previous year. She accepted the offer, thinking it was a business trip, but found out that the “party” was a swinger's party.… On the ride home, the boss man explained that she should keep quiet if she wanted to hold onto her job. (U9)

Here, managerial authority became a mechanism for sexual coercion. The manager's power to dismiss employees enabled him to threaten his employee into silence, demonstrating that the power bestowed on managers by the organizational hierarchy can contribute to sexual harassment going unchallenged. The result was that the manager was able to harass another female employee the following year.

Women also described being disbelieved when they reported managerial harassment; “my supervisor asked me in the break room if I had saved myself for my wedding night. When I reported him he denied it and I was disciplined for making false allegations” (U10). In a case where it was one employee's word against another, the organization chose to believe the supervisor. Though there could be confounding reasons for this, the woman attributes the disciplinary penalties she received to be a direct consequence of his denial. Her perception that his word has more influence within the organization is likely to deter her from reporting harassment in the future. The lack of further investigation conveys the message that one's position in the organizational hierarchy determines their influence and credibility when it comes to matters of workplace conduct. Rather than valuing employees equally, one's occupational role is perceived to be indicative of the likelihood of repercussions. As such, those in powerful positions are able to behave inappropriately without fear of consequences while junior employees are rendered silent if they fall victim to sexual harassment in the workplace.

## The Customer Is Always Right

The second subtheme, *the customer is always right,* concentrates on comments from women working within the service industry who had to tolerate sexual harassment by customers. The comments demonstrated that overt verbal harassment is commonplace within the service industry.

I’ve been sexually harassed at work multiple times by customers. I’ve had one gentleman ask me how my ovaries were working and tell me “thanks for showing me your gals”.… And another tell me that … he wanted to take me home and take advantage of me. Every time I’ve complained, I’m told “oh that's just so and so, that's just how he is”. (U11)

By claiming that *“that's just how he is”* the manager suggests to her employee that sexual harassment is an intrinsic characteristic of particular customers, which implies that it is beyond the organization's responsibility and power to tackle it. However, the repeated harassment of this woman by multiple different customers suggests otherwise. By failing to challenge harassing behaviour, the organization passively endorses poor treatment of their staff. This confirms to customers that they occupy the dominant position within the environment which they can exploit to verbally harass female staff members.

In other cases, organizational policies actively sustained the imbalance of power between customers and staff:

He later complained to corporate (kept referring to us as little girls in the complaint) and we got written up for being rude to a customer.… It's pretty routine for male customers to lose it when we aren’t nice enough, don’t flirt back (even when their wives are 3 feet away), we back away when they step too close … our company just demands an automatic write-up whenever a customer complains to corporate, no matter what actually happened. We can make no defense. (U12)

The complaints procedure silences the woman, as she cannot make a defense, and awards the customer unlimited power not only to harass her but to punish her if she fails to entertain his behavior. As such she is forced to endure and humor harassment from customers in order to avoid facing professional repercussions, completely stripping her of her power to escape harassment while at work.

## Male Domination

The job-gender context had a significant impact on the power dynamics within an industry which in turn had consequences on women's experience of sexual harassment. The subtheme, *male domination,* includes comments from women who worked in predominantly male organizations where organizational norms tolerated or supported sexual harassment. In multiple comments, women described how they were expected to adjust to male styles of communication and behavior. For instance, a female correctional officer in an all-male prison described how:

When I was walking to my post one day an inmate screamed at me “bitch, I’ll beat your pussy so hard it’ll be put you in the hospital.” I called a unit code because to me, that is harassment. Violent sexual harassment. But because I worked in a male prison I was supposed to let it go in one ear and out the other. (U13)

The male-dominated environment is used to justify sexual harassment with the onus being placed on the woman to accept and cope with verbal abuse. The unit code's response demonstrates that they are accustomed to hostility toward women. Their advice to *“let it go in one ear and out the other”* because of the all-male environment implies that the gender ratio of an organization determines its norms. Despite being prisoners, the men are given the power by virtue of their majority and their behavior is deemed to be unchangeable. As such, the minority is expected to adapt to fit in with male communicative norms.

While in other organizations sexual harassment tended to be perpetrated by an individual, some women working in male-dominated industries experienced sexual harassment from groups of male employees.

I worked as the office manager for an industrial insulation company and I was the only woman. I had just come back from maternity leave. I was worried about my milk supply being negatively affected so I went into the bathroom to pump for about 15 mins every 2 hours. All of the men in the office would stand in the break area (right in front of the bathroom door) and make baby crying noises to make fun of me … they would make crying noises every time they passed my desk in hopes that I would leak through my shirt. They would also make comments about how much larger my breasts were since having a baby. (U14)

Here, male “locker-room culture” has become rife in the workplace. The public and group nature of the harassment is both intimidating and humiliating for the victim, who is isolated by her gender. As in the male prison, the organizational norms surrounding acceptable workplace behavior have been affected by the gender ratio of the employees. The act of breastfeeding has become sexualized by the males in the office. Rather than treating her as a valued, equal colleague, she is being objectified by their sexualized banter. While men's lack of insight into the breastfeeding experience may have contributed to their insensitivity, the professional conduct norms within this comment were affected by the job-gender context.

## Dominance via Isolation

Even when men were equal to women in terms of professional position, women experienced male colleagues who engineered situations so as to increase their dominance over women. For instance, many women described how men isolated them before sexually harassing them.

When the last person left, the drunk co-worker stood up, pinned me against my bed, tried kissing me and feeling up my shirt. When I finally got him off, I walked over to the door to kick him out. Turns out, he locked the door so no one would be able to get back into my room. (U15)

By locking the door, the perpetrator has deliberately orchestrated a situation minimizing the likelihood of witnesses to the incident. In doing so he heightens his power, both in the immediate situation and in the aftermath. Consequently, the perpetrator *“went around telling everyone we slept together. I was very lucky to have two male friends who felt so bad for leaving and believed me when no one else did” (U15).* Without evidence, the perpetrator can lie about the incident and subject his victim to further humiliation. Despite it being one word against another, the majority of staff members believed the perpetrator which results in the woman being further harmed by the incident as she becomes the subject of rumor among her colleagues. Despite this, she feels *“lucky”* to have been believed by two of her friends, demonstrating that women expect not to be believed when they are sexually harassed.

Other women had similar stories of men sexually harassing them while they were isolated. One woman described how “the creepy maintenance guy would ask me if I wanted to see his dick… (usually with a nice crotch grab). Of course, it was always when no one was around” (U1). Using the phrase “of course” demonstrates that she is aware that isolation makes her vulnerable to sexual harassment. However, the fact that it “always” occurs suggests that she feels she cannot stop it from happening. The implication is that without witnesses she cannot expect to be believed. There is a tone of resentment within her comment at the fact that the maintenance guy can engineer the situation so as to increase his power and render her powerless to stop him.

## Knowledge Is Power

Within this research, the majority of the online commenters were young and inexperienced when they were sexually harassed. The subtheme, *knowledge is power,* includes comments from women who felt that their youth and inexperience affected their response to sexual harassment. While comments from older or more senior ranked women generally resulted in the victim challenging or reporting their harasser, younger, less experienced women often felt uncertain as to whether their experience was sexual harassment. On other occasions, they had recognized that the behavior was harassment but they did not have the confidence to tackle it. These comments suggest that one's power increases with age and gaining worldly and job-specific experience. For example, one woman described an incident that occurred while she was getting a lift home from babysitting, aged *“13 or 14.”*

I had noticed him staring at me—specifically my chest—earlier, but I didn’t think much of it … he asked me where I went to school (normal), what I did outside of school (a little weird but eh) and if I had a boyfriend (creepy). I, being an innocent lil nerd … remember feeling flattered that he asked me so many questions about my life and seemed to listen to my response (although thinking back I now realize he was looking at my boobs, not my face). (U16)

The woman has come to understand her experience as she has aged but at the time her harasser was able to take advantage of her naivety to “check out” her body and invade her privacy without being challenged. Another woman described a similar experience which she had while working at an ice cream shop for the summer; *“an old man with a cane comes in asks me for some samples … he asked me to feed it to him. Well naïve young me fed him the sample and he starts groaning and making eye contact” (U17).* Her inexperience meant she did not recognize the signs of inappropriate behavior when he asked her to feed him samples. This results in her being exposed to further harassment and humiliation as she naïvely tried to be helpful.

Even in cases where young victims of sexual harassment did recognize that the behavior was inappropriate, they were often unable to tackle it assertively. For example, one woman described how she was:

approached by an expert in the field who was … sixty? Maybe closer to seventy? We (me and the other students) had been encouraged to network … so I began discussing my educational background and professional interests … he began offering some very uncomfortable compliments and asked if I wanted to go dancing. I obviously didn’t, and had to spend the remainder of the conference repeatedly explaining that I wasn’t interested without offending him, and thus potentially burning myself. It was humiliating and frustrating. (U18)

Despite her discomfort and embarrassment, the victim prioritizes protecting her harasser's ego. As such, she suffers greater emotional distress as a result of entertaining his harassment, which manifests in her feeling humiliated and frustrated. Rather than feeling empowered to challenge his behavior, she feels paralyzed by her fear for her career, which negatively impacts her self-worth. His experience and her infancy in the working world compromised her capacity to stand up to harassment.

## Money Makes the World Go Round

In the final subtheme, *money makes the world go round,* women described how their financial position informed their decision of whether to report sexual harassment. Women were aware that reporting harassment could lead to their employer imposing financial sanctions upon them. As such, harassers held greater power over women who could not afford to lose their job or be demoted. These women often chose not to report the incident. For example, one woman explained that *“my boss began telling us that he thinks women are bad at things because they’re too emotional … I feel bad about not saying anything, but I gotta pay my rent” (U19).* Financial need meant she chose not to challenge her boss’ hostile attitude toward women despite the fact that she knew his behavior was inappropriate. She implies that challenging her boss may have led her to lose her job. The fact that she couldn’t see a safe place within her organization to report sexual harassment alludes to a problem with the organizational climate. Due to the lack of appropriate channels to support employees who face sexual harassment, she is coerced into silence by her dependence on the job for financial survival.

Other women described how their financial position was consciously considered when they responded to sexual harassment:

I felt so harassed and unsafe that I would dread going to work every day.… My husband was furious and I had to convince him not to take any drastic action so I could be sure to have a good reference if I needed to find another job. We had a long conversation and looked at our finances and decided the extra money wasn’t worth the emotional distress so I ended up quitting. (U20)

The power of the organization to dictate her professional prospects through the provision of a reference means the victim feels that it would be impossible to report the harassment. It is clear that she believes she would be unsupported by her organization if she spoke out against her harasser. She is left with the decision of whether to endure the harassment or leave. This decision is influenced by her financial position, demonstrating that money can both enable or prevent women from escaping a harassing environment.

Regardless of whether women chose to stay or leave the organization where they were being harassed, the fact that many women within the sample believed they would be punished for reporting harassment demonstrates a major problem within organizational cultures. Women are being forced to alter their lives to survive within a workforce which is hostile to their gender. In the final theme, the effect of organizational responses on women's experiences of sexual harassment are further explored.

### It's in the Culture

This theme explores the significant impact which ineffective organizational responses had on women's experiences of sexual harassment. Though many of the women had chosen not to report harassment there were also numerous women who had done so. It was very common for organizations to have responded negatively to these women, though this manifested in multiple ways.

## Victim Blaming

The subtheme *victim blaming* included cases where organizations held the victim responsible for harassment. One woman described how a customer:

tells me I’m turning him on and I owe him at least a kiss for that … he grabs the chain on my necklace, pulls me over the counter and plants a kiss right on my lips … I told my boss when she returned, turns out he was a friend of hers. She proceeded to bring it up every time he came up in conversation, saying that I made such a big deal out of it that I obviously wanted it and clearly must have encouraged him or led him on in some way. (U21)

The manager's response perpetuates the stereotype that women “ask for it” which diminishes the perpetrator's responsibility for the harassment and suggests that the victim was complicit in the incident. By humiliating her employee *“every time”* the perpetrator is mentioned the manager actually prolongs and worsens the harassment. By suggesting that female provocation is a justification for harassment, the manager conveys to her employee that the perpetrator's sexual urges are valued more than her right to consent. Other women also worked for organizations who claimed that they had “asked” to be harassed. One organization used an employee's appearance to justify harassment:

the TOP DOG on location walked over to me, pulled me aside and told me he thinks about me when he's sleeping with his wife … I told my boss who said “that's the industry and when you look like that you should expect it.” (U22)

The boss implies that to avoid harassment, she should change her appearance. Rather than holding the perpetrator to account, this perspective contributes to creating a culture where women are responsible for sexual harassment.

Multiple women were accused of overreacting or exaggerating by their employers. For example:

my first year teaching I was sexually harassed quite regularly by a group of male students. It got to a point where I was uncomfortable walking down the hallway from my classroom to my office. I approached my principal, expressing my extreme discomfort … his exact words were, “Before we do anything, we need to figure out just how serious this is.” I was crushed. I was made to feel like I was overreacting and that involving him was an inconvenience. (U23)

The principal trivializes the commenter’s experience which leaves her feeling unsupported by the school. He conveys the message that her well-being is not important enough to trigger a *“serious”* response. She is left feeling *“crushed”* by the principal's reaction, demonstrating that an organizational response can worsen the psychological consequences of sexual harassment by decreasing the victim's self-worth. This response suggests that the organizational climate within the school is tolerant of students who sexually harass their teachers. Fostering this culture is likely to increase cases of harassment but decrease the number which are reported because the school community learn that harassment is tolerated by those in power.

Other women described organizations who accused them of lying about harassment. One woman reported that a co-worker was making rape jokes toward her:

When I reported it, the store manager told me to look for a new job and maybe I needed to get my mental health evaluated. (This happened to other girls who worked there.… My store manager promoted the coworker who sexually harassed all the girls and cut my hours because I filed a complaint and spoke up. (U24)

The manager uses the victim's mental health as a means of undermining the legitimacy of her report. The victim's situation is worsened by reporting the incident as she faces social and professional repercussions. By punishing victims and promoting perpetrators, the organization creates a culture where women are less inclined to report sexual harassment. Further evidence of the systemic nature of the issue is conveyed by the woman when she explains that this happened to multiple *“other girls.”*

## That's Just the Way It Is

Even when women were not directly blamed for sexual harassment, it was common for organizations to normalize it. These women, many of whom were working in the hospitality industry, felt that sexual harassment was considered acceptable by their organizations and indeed by the industry as a whole. One woman explained:

Sexual harassment is part of the culture in every restaurant I’ve worked for. The kitchen staff openly comment on female servers and female managers. The saddest thing is that it's never properly addressed ’cause cooks always have more job security that a server. I’ve even had a manager tell me straight up anybody can take that coke to that table but not everyone can make an omelet. (U3)

The managerial response conveys that not all employees are valued equally. Those whose role garners more value are allowed to behave inappropriately without facing repercussions. An organizational hierarchy among the staff has been created by the organization, mirroring that normalized throughout the industry, which means that sexual harassment by some members of staff is permitted. The open manner in which sexual harassment is discussed with this woman demonstrates how entrenched it has become in the organization. Other women reported similarly open discussion of sexual harassment at work:

Me: Can I sit with a member of the sales team to hear how they interact with customers? Manager: oh yeah, you can sit with XX, but just make sure to wear a turtle neck or a high cut shirt, he will stare and will get real close. (U25)

Sexual harassment by certain staff members has become routine in this organization exemplified by the mundane way in which the manager advises the staff about managing harassment. Providing victims with strategies to avoid harassment, rather than tackling the perpetrator, allows sexual harassment to become integrated within the organization's functioning. Returning to a point addressed earlier, this kind of organizational response feeds the stereotype that women “ask for” sexual harassment through the way they choose to dress and again reinforces women's responsibility for dealing with sexual harassment.

There were further examples of organizations having become numb to sexual harassment. In one case, a customer was harassing a restaurant hostess who *“went to her female manager and said, “That guy just tried to kiss me and he kept offering me money!!” Her female manager didn’t even blink an eye. She just said, “He's a little drunk, that's all” (U26).* The term *“didn’t even blink an eye”* demonstrates that the woman perceives the manager to be unperturbed by sexual harassment. This suggests that it is a regular experience within the restaurant. The fact that the manager has developed justifications for sexual harassment, such as intoxication, further demonstrates the normalization of sexual harassment. By justifying the behavior and failing to act sexual harassment of staff becomes a self-fulfilling issue. As it perpetuates its normalcy is reinforced and the less likely it is to be tackled.

## Discussion

Workplace sexual harassment of women has been ongoing for generations and efforts to tackle it have been fraught with challenges due to the culture of silence which surrounds it (Baum, 2019). Researchers have struggled to gain an insight into the causes and conditions surrounding workplace sexual harassment because of the huge number of unreported cases. However, in recent years, social media movements such as #MeToo have opposed the taboo surrounding sexual harassment by creating online communities for women to openly voice their experiences (Karami et al., 2019). This study aimed to capitalize upon this opportunity to study women's experiences of sexual harassment in a deeper, more candid and natural way. Notably, this study harvested data from an online thread asking women about their wildest workplace experiences. While sexual harassment was not the specified theme, 86.5% of the threads detailed sexual harassment, highlighting the pervasiveness of this within the workplace context.

The findings of this study supported and built upon the work of previous researchers in the field (Fitzgerald & Cortina, 2018; Karami et al., 2019; Walker, 2018). The results suggest that women are experiencing sexual harassment regularly, from multiple perpetrators, and in different occupations. Rather than being an isolated incident involving deviant individuals, women are experiencing sexual harassment as endemic within the workplace, underpinned by an imbalance of power between men and women and sustained by ineffective organizational responses. Male dominance within the working environment is making women vulnerable to experiencing harassment, while organizational responses are rendering women powerless to tackle it. Where a “double dominance”—numerical dominance and normative dominance—exists (Gruber, 1990), harassment of female staff often takes place unchallenged. Women in this study were frequently told by their organizations that sexual harassment is part of the job or that they asked for it. In some cases, they were even punished for exaggerating or making false allegations. These toxic organizational responses are having significant consequences for working women. For many women the response from their organization left them with the perception that harassment is normal and thus they expressed a lack of confidence over whether their experience “counts” as harassment. Other women chose to endure harassment despite knowing it is wrong because they could not afford to face the negative repercussions of reporting it. In some cases women were so distressed that they chose to leave a particular industry, or the workforce in general, because they could not cope with the harassment. Without action, the modern workforce risks losing valuable female contribution, while further entrenching male dominance of major industry sectors. Indeed Dainty et al. (2000), taking a radical stance, suggested that women should refuse to enter the construction sector until the clear barriers to their career progression (namely overt and covert discrimination and the strategic use of structural systems by males to block progress) are made. Ultimately, industries and organizations with greater gender balance, particularly at leadership levels, are needed to effectively unsettle the double dominance (de Haas & Timmerman, 2010). Indeed, it has been suggested that a 20%–30% threshold of a population is needed for social change, evidenced by the reform of the military sexual assault protocol when the US Senate reached 20% female and the downfall of the casting couch culture when women producers in Hollywood reached 25% (Newton-Small, 2017).

The naturalistic nature of the data used in this study means that it makes a unique contribution to the literature surrounding workplace sexual harassment. Since the data was initially created without research purposes in mind, the Buzzfeed users who commented on the thread were unaffected by any actions made by the researcher. This, and the fact that their responses were anonymous, means that they are less likely to have been affected by social desirability concerns, or fears that their responses will be shared with their employers. Furthermore, writing within an online community of women with shared experiences may have encouraged greater detail within their comments. Maintaining detachment from the women commenting also had benefits for the researchers. Having never met the women and only knowing the information they provided within their comments reduced the possibility of the analysis being impacted by any unconscious biases the researchers held. This allowed the data to speak for itself without preconceptions about the individual clouding interpretation.

Although detachment between the researchers and the women commenting is a valuable research asset, the lack of demographic information about the commenters is a potential study limitation. As with other internet-mediated research, the researcher had to rely upon cues within the comments to infer details about the commenters gender and age (Karami et al., 2019). While it appeared that all were women, it is possible that they were not, as the researcher could not control who commented on the thread. The need for internet access, and knowledge of Buzzfeed as a forum, means that contributions may have been more likely to have come from younger individuals who engage with this medium more regularly (Sadri, 2018). Finally, inequalities are often influenced by intersecting characteristics, for example, gender, sexuality, and race (Rosette et al., 2018), and in this study, no information on sexuality or race could be inferred. Thus the important issue of how women with differing intersectional identities are affected could not be explored.

Despite the limitations, the findings have important implications for organizational policies and future research. The results indicate that sexual harassment stems from toxic organizational cultures, which unevenly distribute power and fail to support victims. As such, they demonstrate that organizations need to implement effective channels of support for victims of sexual harassment. Furthermore, organizations need to develop more respectful working cultures by instilling organizational norms which are intolerant of sexual harassment, such as codes of ethics (Poulston, 2008). A key element of this process will be employee education and adequate training. Employees need to be clear on what constitutes sexual harassment; how to challenge it when they witness it; how to avoid becoming a perpetrator of it; and how to access the necessary support if they become a victim. Focusing on accountability, action, and transferability of training, such as rewarding reporting, is a key part of transforming culture (Sachdev et al., 2019). Developing a clearer anti-harassment position within organizations is integral to reducing its prevalence, as currently women are working within environments where perpetrators can harass them totally unchallenged by their organization. Visibility and overt managerial opposition to harassment have been shown to lead to lower rates of harassment in the hospitality sector (Poulston, 2008). Industry-specific training is likely to be more effective than generic training as it can address the industry-specific culture and climate (Dawson et al., 2021). Indeed, training needs to take a multifaceted approach, encompassing the environmental (e.g., protecting workers from unsafe tasks/environments/customers), the administrative (e.g., implementing strict and legal policies, guidelines), and the behavioral (e.g., interventions, education, free counseling, and support), so that organizations can more effectively reduce sexual harassment (Gutworth & Howard, 2019).

In terms of research, this study has demonstrated the value of internet data for investigating victims' experiences of sensitive issues. Social media and the internet are being used continuously as platforms for debate, particularly during the ongoing coronavirus pandemic, and so this type of data could be used to explore a range of other issues, such as LGBTQI + concerns. At the time of writing, a new media outburst has emerged in the wake of the disappearance and murder of Sarah Everard in the UK. The tragedy has sparked online conversations about the prevalence of street harassment. The conversation suggests that the acceptance of sexual harassment may exist at a level beyond that of organizations and within the structures of an oppressive society. This warrants further investigation by future researchers.

For workplace sexual harassment specifically, future research should explore men's perspective on the issue. It is pertinent to understand how men define and understand sexual harassment as results from this study indicate that men are failing to recognize their own, and others, harassing behavior. An understanding of men's perspective could help education programs in designing and targeting their interventions. It would also be valuable for researchers to conduct a longitudinal study that follows working women throughout their careers. This would help to discover whether women's experiences change over their working life, as well as indicating whether organizational climates are changing as a result of increased public discussion about sexual harassment thanks to movements such as #MeToo.

This study hopes to have contributed to the understanding of the pervasive yet normalized nature of workplace sexual harassment and the consequences it has for women's personal and professional prospects. It is a complex issue that exists beyond the level of the individual and within the foundations and functioning of organizations. However, there is hope that the growing number of women's voices being heard through online platforms can contribute to changing the climate of individual organizations and wider society, championing intolerance toward the sexual harassment of women at work and in their daily lives.
